# Propensity score analysis with missing data using a multi-task neural network

**DOI:** 10.1186/s12874-023-01847-2

**Published:** 2023-02-15

**Authors:** Shu Yang, Peipei Du, Xixi Feng, Daihai He, Yaolong Chen, Linda L. D. Zhong, Xiaodong Yan, Jiawei Luo

**Affiliations:** 1 School of Intelligent Medicine, Chengdu University of Traditional Chinese Medicine, Chengdu, China; 2 West China Biomedical Big Data Center, West China Hospital/West China School of Medicine, Sichuan University, Chengdu, China; 3 Department of Applied Mathematics, Hong Kong Polytechnic University, Hong Kong, China; 4 School of Public Health, Chengdu Medical College, Chengdu, China; 5 Institute of Health Data Science, Lanzhou University, Lanzhou, China; 6 Biomedical Sciences and Chinese Medicine, School of Biological Sciences, Nanyang Technological University, Singapore, Singapore; 7 School of Chinese Medicine, Hong Kong Baptist University, Hong Kong, China; 8 School of Economics, Shandong University, Jinan, China

**Keywords:** Observational study, Propensity score analysis, Neural network, Multitasking learning, Causal effect estimation, Inverse probability weighting

## Abstract

**Background:**

Propensity score analysis is increasingly used to control for confounding factors in observational studies. Unfortunately, unavoidable missing values make estimating propensity scores extremely challenging. We propose a new method for estimating propensity scores in data with missing values.

**Materials and methods:**

Both simulated and real-world datasets are used in our experiments. The simulated datasets were constructed under 2 scenarios, the presence (T = 1) and the absence (T = 0) of the true effect. The real-world dataset comes from LaLonde’s employment training program. We construct missing data with varying degrees of missing rates under three missing mechanisms: MAR, MCAR, and MNAR. Then we compare MTNN with 2 other traditional methods in different scenarios. The experiments in each scenario were repeated 20,000 times. Our code is publicly available at https://github.com/ljwa2323/MTNN.

**Results:**

Under the three missing mechanisms of MAR, MCAR and MNAR, the RMSE between the effect and the true effect estimated by our proposed method is the smallest in simulations and in real-world data. Furthermore, the standard deviation of the effect estimated by our method is the smallest. In situations where the missing rate is low, the estimation of our method is more accurate.

**Conclusions:**

MTNN can perform propensity score estimation and missing value filling at the same time through shared hidden layers and joint learning, which solves the dilemma of traditional methods and is very suitable for estimating true effects in samples with missing values. The method is expected to be broadly generalized and applied to real-world observational studies.

**Supplementary Information:**

The online version contains supplementary material available at 10.1186/s12874-023-01847-2.

## Introduction

In observational studies, propensity scores are increasingly used to control for confounding [1, 2]. When the observed baseline characteristics are sufficient to correct for confounding bias and the propensity model is correctly constructed, a conditional exchange can be conducted between subjects with the same propensity score [3, 4]. Observational studies usually inevitably have covariates with missing values. Currently, estimating the propensity score in the presence of missing values is a challenge for studying causality [5–8]. Common approaches to dealing with missing values in propensity analysis include full-case analysis, adding missing indicator variables to the propensity model, and multiple imputation [9–11]. Unfortunately, these methods are inherently flawed. For example, the missing indicator method introduces new biases [12]. There are studies using machine learning methods to replace traditional logistic regression [13–17]. However, they do not address the propensity score misestimation problem caused by overfitting. In contrast to hand-crafted models [18], neural networks can automatically learn interactions between variables. A multi-task neural network is a network structure with multiple outputs. It has been widely used in the medical field. With a multi-task neural network, propensity score computation and missing value filling can be performed jointly. By optimizing the global objective function, overfitting to the propensity score calculation task can be prevented, while the estimation problem of missing value [19] is effectively solved. This study develops a new pipeline for calculating propensity scores in samples with missing values based on a multi-task neural network. To evaluate the accuracy of our model in estimating the true effect, we conduct experiments on simulated and real-world data separately, and compare our method with traditional methods.

## Data and methods

### Propensity score

In a study, individual subjects may have multiple covariates. Propensity scoring is a way of simplification multiple covariates [20]. It condenses multiple covariates into a single variable (propensity score), whose meaning is the conditional probability of being assigned to the experimental group depending on the covariates [21]. A propensity score can be viewed as a function of the original multiple covariates, so the propensity score includes information about these covariates. Rosenbaum and Rubin demonstrated that the propensity score *e*(*X*) can be used to balance the distribution of a covariate between experimental and control groups when the covariate X meets the strong negligibility assumption [3].

$$e\left({X}_i\right)=\Pr \left({T}_i=1\left|{X}_i\right.\right)$$

### Propensity score estimation

In complete data, logistic regression is the most commonly used method for estimating propensity scores under the conditions of binary treatment or exposure [22]. The propensity score is calculated by performing binary regression on covariates (i.e. potential confounders) by treatment or exposure indicator variables, which can be written as:

$$\textrm{logit}\left({p}_i\left(T=1\right)\right)={X}^{\prime}\beta, i=1,2,\dots, n$$

where, *X*^′^ = (1, *X*_1_, *X*_2_, …, *X*_*K*_), *β* = (*β*_0_, *β*_1_, *β*_2_, …, *β*_*K*_)^′^, *K* is the number of covariates and n is the number of observations. An individual’s propensity score can be estimated as

$${p}_i=\frac{{\textrm{e}}^{X_i^{\prime}\beta }}{1+{\textrm{e}}^{X_i^{\prime}\beta }}$$

In many situations, logistic regression may not be the best choice when estimating propensity scores. We assumed that the log probability of exposure was linearly related to covariates when using logistic regression to estimate exposure probabilities. However, this assumption is not always true. Logistic regression cannot estimate propensity scores accurately when covariates interact with each other or when covariates and treatments are not linear. To solve the inherent problem of logistic regression estimation of propensity scores, some studies substitute machine learning algorithms for logistic regression. These include decision trees, random forests, Naive Bayes, support vector machines, etc. [13–15, 23, 24] It is claimed that these methods can provide a more accurate estimate of propensity scores. Nevertheless, these conclusions have not been validated by systematic simulation studies.

### Missing data

In realistic observational studies, individual covariates may have large amounts of missing data, which may lead to both loss of efficiency and biased estimates. Based on the degree to which confounding factors are related to outcome and exposure, the magnitude of bias varies.

#### Type of missing data

There are three types of missing data depending on the mechanism of missing: missing completely at random (MCAR), missing at random (MAR), and missing not at random (MNAR) [25, 26]. MCAR refers to missing data when a random subset of the study population has the same probability of being missing. In contrast to MCAR, the term MAR is counterintuitive. MAR occurs when the probability of missing is dependent only on the observed information. Missing data are denoted by MNAR when their probability depends on the unobserved data, such as the observation value itself.

#### Methods for handling missing values

Complete case analysis is the easiest way to deal with incomplete confounding data, which restricts the analysis to cases where all variables are complete. If the absence of covariates is independent of treatment and outcome, then this approach provides unbiased estimates of group effects. Another simple method is the missing indicator method [27]. Before incorporating confounding into a propensity score model, add a “missing” category to partially observed categories. Continuous confounders are set to a specific value, such as 0, and both the confounding factor and missingness indicator (a variable that indicates whether the variable is observed) are included in the propensity score model. In many cases, this approach leads to biased results. Missing pattern analysis is a generalization of the missing index method. This method is used when all individuals are grouped together according to different missing patterns. Then, propensity scores are estimated in each group separately. As a practical matter, this method fails when the number of participants with missing patterns is lower than the number of observed covariates. It usually occurs when there are a lot of missing patterns in the data. Multiple imputation is a method of using chain equations to impute incomplete data, in which the missing covariates are imputed with plausible values based on the predicted distribution of the missing covariates in a set of observed data many times to create complete datasets [28, 29]. We used MICE (version 3.3.0) in R (version 3.6.3) to perform multiple imputation. A Bayesian linear regression was used for the mice model. It is commonly used when covariates and outcomes are continuous. Other parameters are set as defaults.

### Inverse probability weighting

Inverse probability weighting (IPW) uses the inverse of the propensity score as weights to create a synthetic sample in which the baseline covariate distribution is independent of treatment assignment [30]. In this study, we use IPW to estimate the true effect. Unlike propensity score matching, IPW uses all individuals in both groups, thus avoiding sample waste. A high level of statistical power was maintained in all cases to detect effects. IPW was more sensitive to erroneous propensity score estimation. This limitation emphasizes the importance of carefully defining model selection before applying propensity score weighting. Multi-task neural networks can overcome this limitation.

### Multi-task neural network

**Fig. 1:**
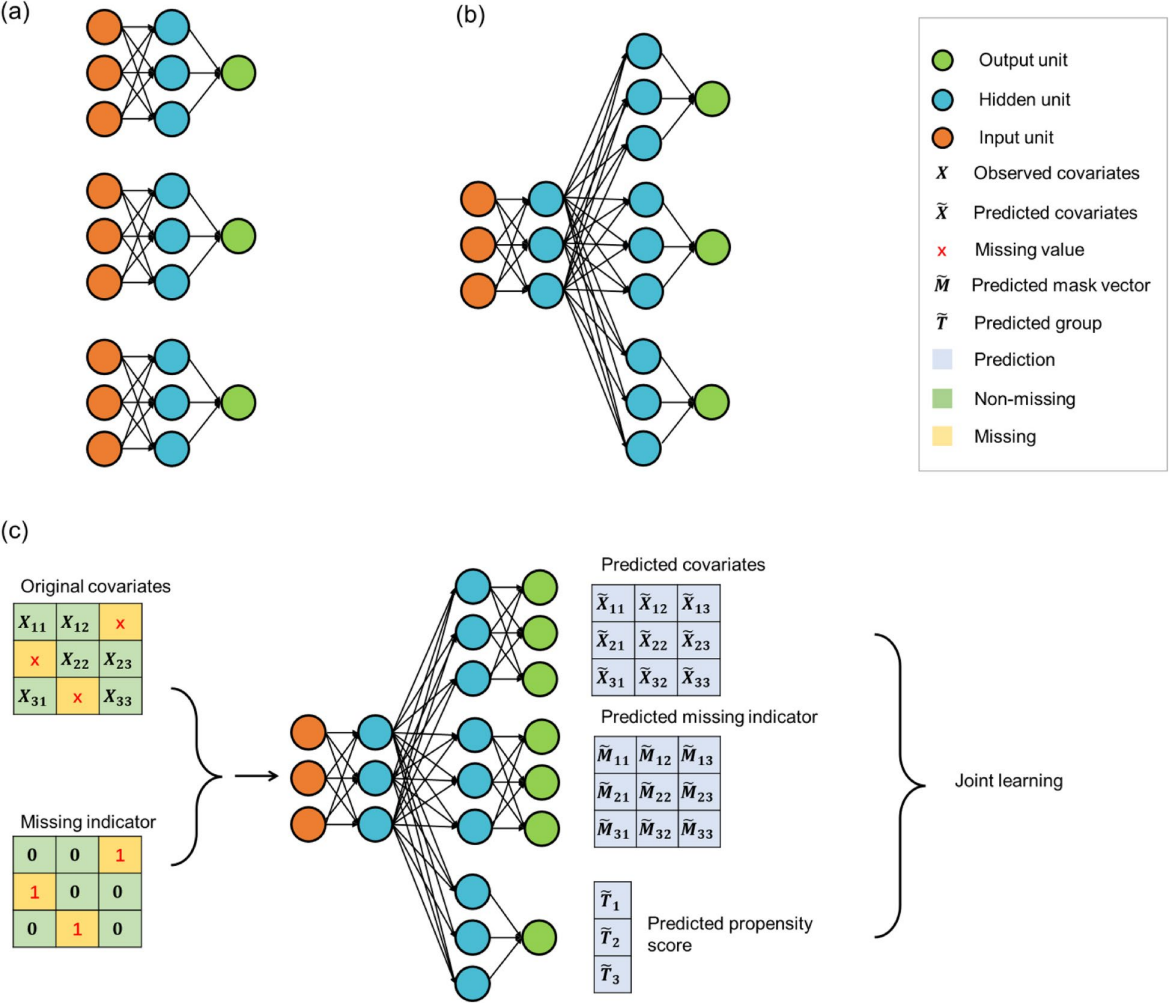
Structure diagram of multi-task neural network

Neuronal networks are excellent function approximators, which can estimate linear and nonlinear functions. It uses data samples with known outcomes as examples for supervised training. In this process, a nonlinear function model is built to predict the output data based on the input data. Figure 1**(a)** shows three independent neural networks. All networks have the same inputs and outputs. Back-propagation is used to train each net separately. There is no connection between the three nets, so the information that one learns cannot help the others. This is known as single-task learning (STL). Figure 1**(b)** shows a single net with the same inputs as those on the left, but three outputs corresponding to the learning task. Each of the 3 outputs is connected to the same hidden layer. Three of the MTL outputs undergo parallel backpropagation. These results share a hidden layer, meaning the internal representation of one task is available for other tasks. The core idea of multitask learning is to share knowledge learned from different tasks and to train them simultaneously.

In this study, we propose a novel pipeline using a multi-task neural network (MTNN) to estimate propensity scores. There are three parts to our task set: reconstructing input covariates, estimating propensity scores, and predicting missing patterns. There is a close relationship between these tasks. The structure of MTNN is shown in Fig. 1. In order to achieve joint optimality across all tasks, the MTNN must correctly learn the relationship between covariates, covariates and absence, and covariates and exposure levels. Through joint learning and sharing hidden layers, MTNN reduces overfitting when estimating propensity scores. The detailed calculation procedure and more information about MTNN training can be found in Supplementary S1. Our tutorial and source code for MTNN are also available on github^1^ so readers can apply our method to real problems and gain a deeper understanding of it. Models for missing value imputation and estimation of propensity scores are determined from the convergence of the objective function. In all experiments in this study, we chose the model for the last epoch after convergence.

### Data

#### Simulation data

We adopted a data simulation generation process similar to that of Choi [7]. Two scenarios were considered, one in which the outcome was treatment-related (effect≠0), and one in which it was treatment-independent (effect = 0). In each scenario, we considered three different deletion mechanisms. First, we generated 2 continuous covariates, *X*_1_ and *X*_2_, for each subject. *X*_1_ follows a normal distribution with mean 0 and standard deviation 1. *X*_2_ depends on *X*_1_.

$${X}_{2i}=0.5{X}_{1i}+{\varepsilon}_i\ \textrm{with}\ {\varepsilon}_i\sim N\left(\textrm{0,0.75}\right)$$

In this way, the standard deviation of *X*_2_ is also 1, and the correlation between *X*_1_ and *X*_2_ is equal to 0.5. The treatment T was generated from the binomial distribution, with the probability for subject *I* to receive the treatment being equal to:

$$\textrm{logit}\left(P\left({T}_i=1|{X}_{1i},{X}_{2i}\right)\right)=-0.8+0.5{X}_{1i}+0.5{X}_{2i}$$

By this equation, about 30% of subjects were treated.

We constructed 2 scenarios:

***Scenario 1:*** the outcome is affected by treatment: we assume, without losing generality, that treatment has an effect of 1 on the subject’s outcome.

$${Y}_i={X}_{1i}+{X}_{2i}+ Trea{t}_i+{\varepsilon}_i,\textrm{with}\ {\varepsilon}_i\sim N\left(0,1\right)$$

***Scenario 2:*** the outcome is unrelated to the treatment.

$${Y}_i={X}_{1i}+{X}_{2i}+{\varepsilon}_i,\textrm{with}\ {\varepsilon}_i\sim N\left(0,1\right)$$

To test the effect of different missing rates on effect estimation in simulated datasets, we preset 7 missing rates, including 0.2, 0.3, 0.4, 0.5, 0.6, 0.7, and 0.8. Missing values in *X*_2_ are generated using three mechanisms:

1. MCAR: In *X*_2_, a random proportion of observations is set to be missing.
2. MAR: The higher the value of *X*_1_, the more likely the value of *X*_2_ is missing. Taking *M* as the missing indicator of *X*_2_, the probability of missing *X*_2_ value is:
  $$\textrm{logit}\left(P\left({M}_i=1\right)\right)={X}_{1i}+C$$
3. MNAR: The higher the value of *X*_2_, the more likely the value is missing. The probability of missing an *X*_2_ value is:
  $$\textrm{logit}\left(P\left({M}_i=1\right)\right)={X}_{2i}+C$$

*C* is a constant used to control the missing rate. As an example, if a missing rate of around 50% is to be controlled, C can be set to 0.

#### Real-world data

The real-world data come from a subset of the data from the treated group in the National Supported Work Demonstration (NSWD) and the comparison sample from the Population Survey of Income Dynamics (PSID). The dataset has been used by many researchers to test the effects of different propensity score analysis methods [31, 32]. There are 614 samples in this dataset (185 treatments and 429 controls). Each person has 9 variables. Table S1 provides more details. Treat is the intervention variable, re78 is the outcome, and the other 7 variables are covariates. Table S2 summarizes the distribution of covariates between different treatment groups. It shows that the distributions of the variables age, race, married, nondegree, re74, re75 differ between groups. Therefore, we need to correct the effect estimates with propensity scores.

Our experiments used the inverse probability-weighted effect size of the propensity score calculated from the complete data as the reference. Simulations were then performed to estimate the true effect under the three missing mechanisms. We made missing values occur in both variables re74 and re75. In each of these variables, missing values were constructed randomly. Similar to the setting we used for simulated datasets, we used 7 missing rate settings for real-world datasets: 0.2, 0.3, 0.4, 0.5, 0.6, 0.7, and 0.8.

1. MCAR: In both variables re74 and re75, randomly selected given proportion of observations are set to be missing.
2. MAR: The missing rate is assumed to be proportional to a linear combination of age and education. These 2 variables were chosen arbitrarily without loss of generality, as there were correlations between the covariates (table S8). To facilitate setting the probability of missing, we normalize the age and years of education so that the mean is 0. Let *M*_1_ and *M*_2_ represent the missing indicators of re74 and re75, respectively, then their missing probability is:
  $$\textrm{logit}\left(P\left({M}_{i1}=1\right)\right)=\textrm{ag}{\textrm{e}}_i+\textrm{edu}{\textrm{c}}_i+C$$

$$\textrm{logit}\left(P\left({M}_{i2}=1\right)\right)=\textrm{ag}{\textrm{e}}_i+\textrm{edu}{\textrm{c}}_i+C$$

(3) MNAR: The higher the value of a variable, the more likely that value is missing. Similar to age and years of education, we also normalize re74 and re75. Then the probability of re74/re75 missing is:
  $$\textrm{logit}\left(P\left({M}_{i1}=1\right)\right)=\textrm{re}{74}_i+C$$

$$\textrm{logit}\left(P\left({M}_{i2}=1\right)\right)=\textrm{re}{75}_i+C$$

### Estimation of the true effect

**Fig. 2:**
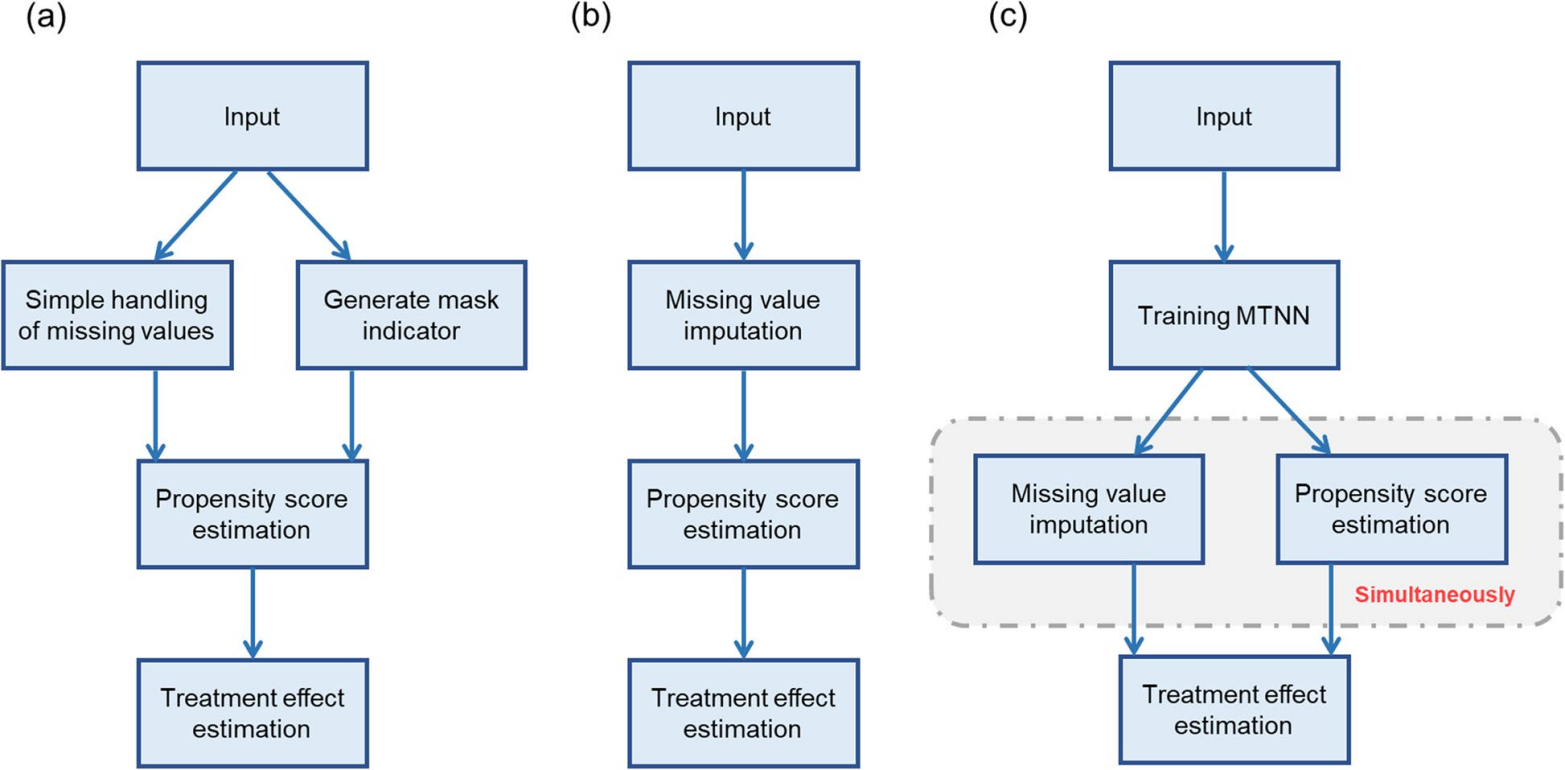
Flowchart of the three methods for estimating effect. a the missing index method; b the multiple imputation method; c multi-task neural network method. MTNN, multi-task neural network

The first step is to deal with missing values in the samples. As MTNN computes propensity scores and imputation values simultaneously, it does not require separate missing value processing. When propensity scores were estimated by logistic regression, multiple imputation and missing indicator methods were used to handle missing values. We estimate propensity scores using age, education, race, marital status, education, and re74 and re75 as covariates. These 7 covariates are also included in the regression analysis used to estimate effect. Lastly, we estimated the effect using an inverse probability-weighted regression analysis of the propensity score, in which subjects receiving treatment were weighed 1/propensity score and subjects not receiving treatment were weighed 1/(1 - propensity score). Figure 2 shows the workflow for estimating the effects of the three methods.

### Evaluation

There are 2 kinds of effects in the experiments with simulated data, and three mechanisms for handling missing values, i.e., 6 scenarios for generating simulated data, and 3 methods for handling missing values. In experiments with real-world data, there are three missing mechanisms, namely three scenarios. For each scenario, the same process of missing value imputation, propensity score calculation, and effect estimation was repeated 20,000 times before evaluating the results of the different methods. Comparisons are conducted based on standard deviations (SD) and root mean square errors (RMSE), which is defined as:

$$RMSE=\sqrt{\frac{1}{n}\sum_{i=1}^n\kern0.1em {\left({\hat{\beta}}_i-\beta \right)}^2}$$

Where $\hat{\beta}$ is the estimate and *β* is the true value.

## Result

### Analysis results on simulation datasets

**Fig. 3:**
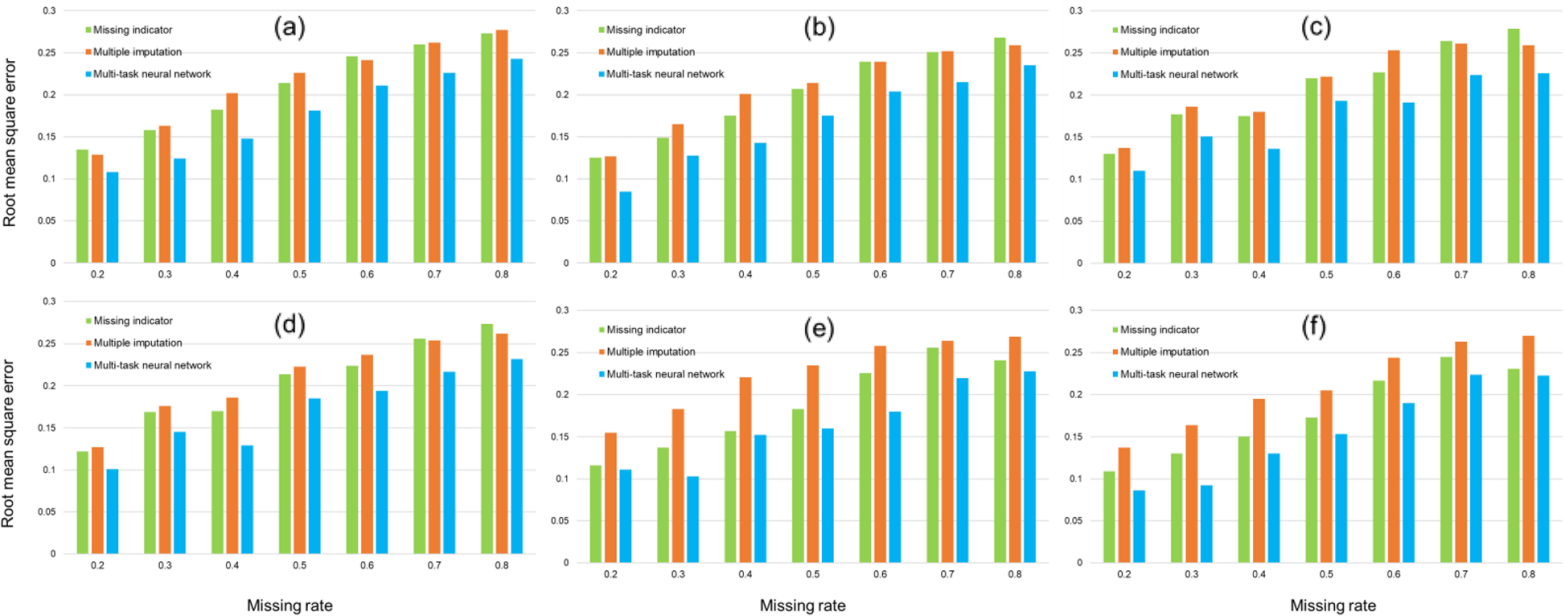
Root mean square error of the true effect estimated by different methods under three missing mechanisms in the simulation dataset. (a), (d) are under MCAR, (b), (e) are under MCAR, (c)-(f) is under MNAR. For **a**, **b** and **c**, the true effect is 0; for **d**, **e** and **f**, the true effect is 1. MCAR, missing completely at random; MAR, missing at random; MNAR, missing not at random

Figure 3 shows the RMSE of the true effect estimates under 2 true effect scenarios and three missing mechanisms. The smallest RMSE for all 6 data scenarios is achieved with MTNN. Thus, MTNN seems to be the best method over the other two. In addition, regardless of the choice of method used, the higher the missing rate, the higher the RMSE. When the missing rate was increased from 0.2 to 0.8, the RMSE for any of the three estimation methods nearly doubled. Table 1, Table S3 and Table S4 present more detailed information on the estimation results for the three methods. In all scenarios of data, we find that MTNN is not only optimal in estimation of true effect deviation, but also that the standard deviation of its estimation results is the smallest. This shows that MTNN provides the most accurate estimation, as well as being more stable than other methods.

**Table 1:** Estimation of the true effect in the simulated datasets using three different methods under the MCAR mechanism

Missing rate	Method	True effect =0			True effect =1		
		Mean	SD	RMSE	Mean	SD	RMSE
0.2	Missing indicator	0.119	0.059	0.131	1.119	0.059	1.12
	Multiple imputation	0.11	0.071	0.128	1.11	0.071	1.112
	Multi-task neural network	0.091	0.052	0.103	1.077	0.055	1.079
0.3	Missing indicator	0.146	0.063	0.157	1.146	0.063	1.147
	Multiple imputation	0.136	0.076	0.153	1.136	0.076	1.138
	Multi-task neural network	0.107	0.057	0.12	1.12	0.052	1.121
0.4	Missing indicator	0.173	0.061	0.182	1.173	0.061	1.174
	Multiple imputation	0.192	0.089	0.209	1.192	0.089	1.195
	Multi-task neural network	0.138	0.05	0.146	1.139	0.058	1.141
0.5	Missing indicator	0.206	0.069	0.216	1.206	0.069	1.207
	Multiple imputation	0.214	0.078	0.226	1.214	0.078	1.216
	Multi-task neural network	0.173	0.058	0.181	1.169	0.06	1.17
0.6	Missing indicator	0.234	0.071	0.243	1.234	0.071	1.236
	Multiple imputation	0.228	0.076	0.239	1.228	0.076	1.23
	Multi-task neural network	0.2	0.064	0.208	1.198	0.06	1.199
0.7	Missing indicator	0.242	0.081	0.254	1.242	0.081	1.244
	Multiple imputation	0.248	0.08	0.259	1.248	0.08	1.251
	Multi-task neural network	0.207	0.078	0.219	1.204	0.071	1.206
0.8	Missing indicator	0.258	0.061	0.264	1.258	0.061	1.259
	Multiple imputation	0.26	0.074	0.269	1.26	0.074	1.262
	Multi-task neural network	0.226	0.053	0.232	1.224	0.057	1.225

SD, standard deviation; RMSE, root mean square error; MCAR, missing completely at random; MAR, missing at random; MNAR, missing not at random

### Analysis results on real-world datasets

We first calculated the propensity score by logistic regression from the complete data, and then used the inverse probability-weighted regression equation to calculate the effect to be 712.743 (Table S**7**). Since the true effect of real-world data is unknowable, we use it as a reference standard to compare the performance of different methods.

**Fig. 4:**
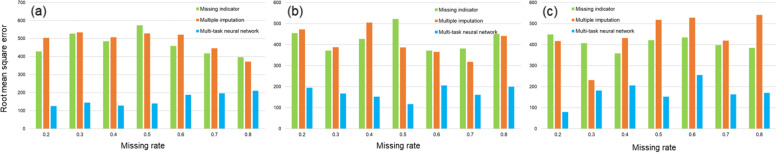
RMSE of the true effect estimated by different methods under three missing mechanisms in the real-world dataset. **a** MCAR, **b** MAR, **c** MNAR

**Fig. 5:**
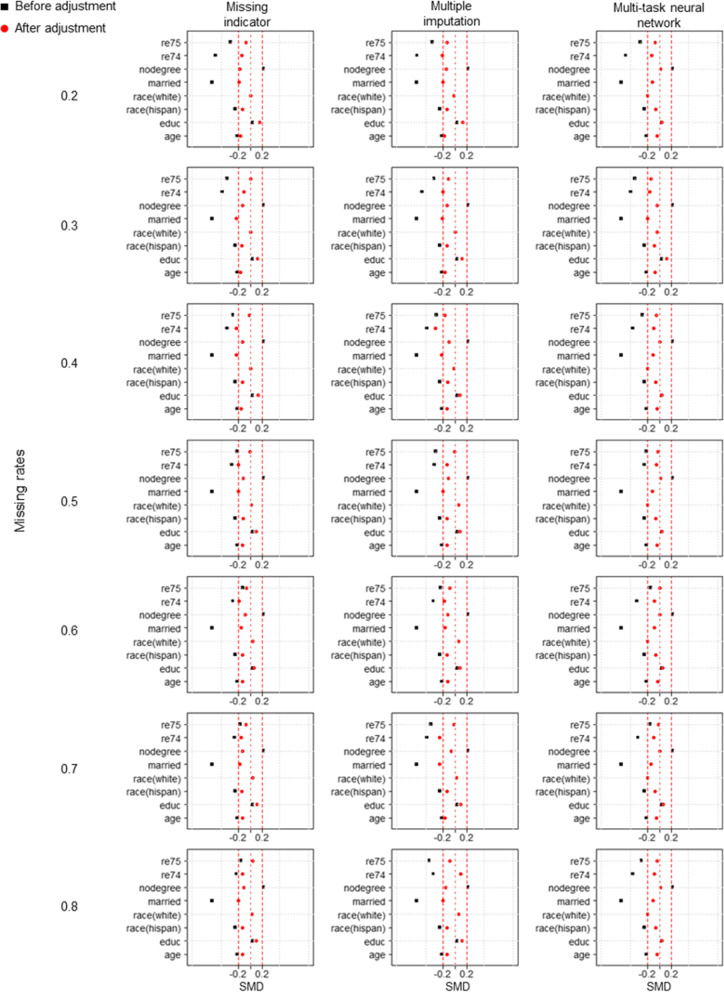
Between-group standardized mean differences under MCAR for covariates adjusted for propensity scores calculated by three different methods

**Fig. 6:**
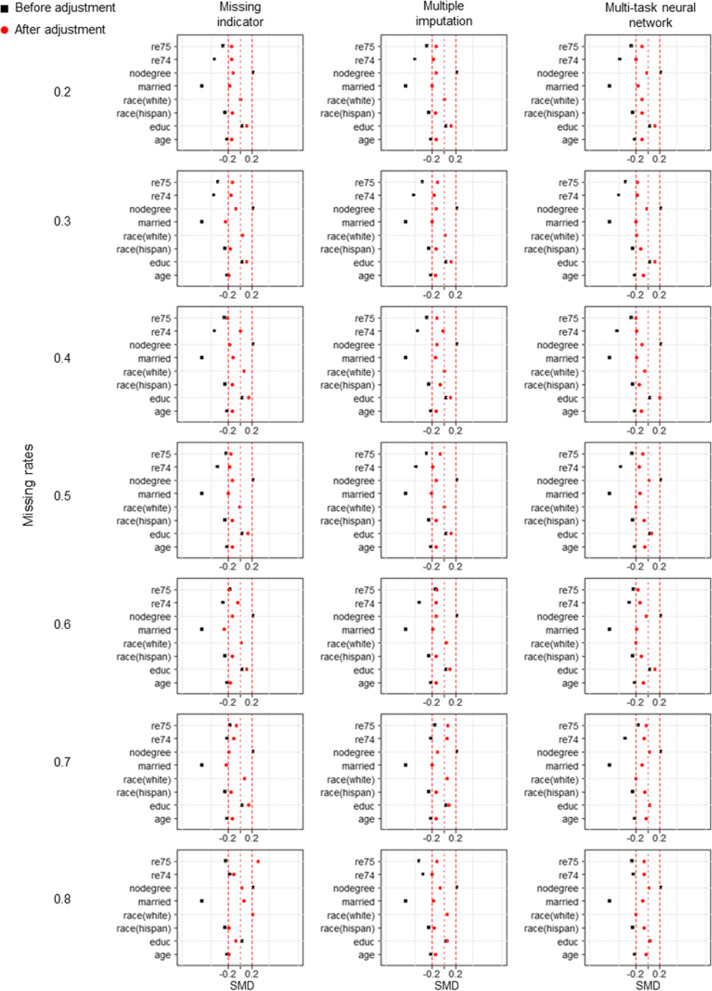
Between-group standardized mean differences under MAR for covariates adjusted for propensity scores calculated by three different methods

**Fig. 7:**
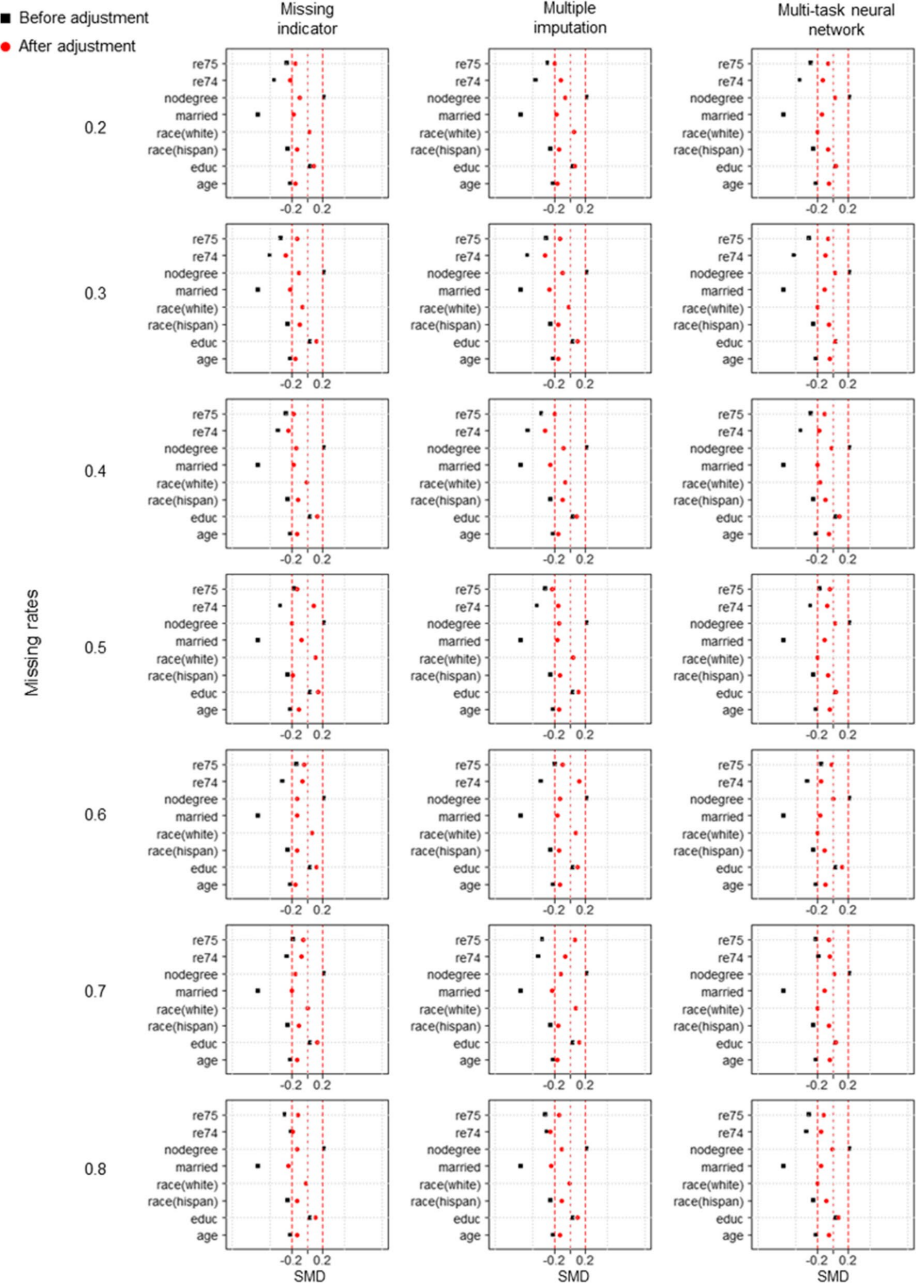
Between-group standardized mean differences under MNAR for covariates adjusted for propensity scores calculated by three different methods

Figure 4 compares RMSE between different methods under three distinct missing mechanisms. According to the analysis results of simulated data, MTNN exhibited the smallest RMSE under different missing mechanisms and missing rates. The difference is that in the real-world dataset, the missing rate is less influential on the RMSE of the estimated result. Table 2, Table S5 and Table S6 provide further details of the estimation results for the various methods. It is clear that the standard deviation of the MTNN estimation results is lower than that of the 2 other methods. Figures 5, 6 and 7 show the between-group standardized mean differences (SMD) of each covariate adjusted by the propensity scores estimated by the three methods under the three missing mechanisms.

**Table 2:** Estimation of the true effect in the real-world datasets using three different methods under the MCAR mechanism

Missing rate	Method	Mean	SD	RMSE
0.2	Missing indicator	352.262	250.792	431.108
	Multiple imputation	198.329	276.932	576.882
	Multi-task neural network	672.620	121.163	121.075
0.3	Missing indicator	395.330	283.958	415.239
	Multiple imputation	403.878	309.952	425.198
	Multi-task neural network	718.292	144.233	136.098
0.4	Missing indicator	316.312	226.881	450.458
	Multiple imputation	277.395	247.175	493.797
	Multi-task neural network	736.417	146.882	140.491
0.5	Missing indicator	240.517	266.086	534.726
	Multiple imputation	341.053	279.437	455.591
	Multi-task neural network	683.922	112.963	110.333
0.6	Missing indicator	339.664	233.459	433.169
	Multiple imputation	171.271	191.980	570.923
	Multi-task neural network	623.39	140.996	160.172
0.7	Missing indicator	323.533	202.261	433.415
	Multiple imputation	318.219	232.415	451.292
	Multi-task neural network	587.779	116.185	166.178
0.8	Missing indicator	328.838	128.502	402.568
	Multiple imputation	395.226	123.355	338.146
	Multi-task neural network	640.485	167.939	174.043

SD, standard deviation; RMSE, root mean square error; MCAR, missing completely at random; MAR, missing at random; MNAR, missing not at random

## Discussion

In this study, we develop a novel method for calculating propensity scores with multi-task neural networks that can calculate propensity scores directly for samples with missing values. On simulated and real-world datasets, we compare the proposed method with two commonly used ones. Under the three missing mechanisms, the RMSE of our proposed method for estimating the true effect is the smallest. In addition, the standard deviation of the true effect estimated by MTNN is the smallest, indicating that it is more robust than the other two methods. While previous studies have demonstrated smaller RMSEs for machine learning algorithms, our study confirms these findings in scenarios with missing values [33–36]. We also found that under lower missing rate conditions, the RMSE of the missing indicator method is better than multiple imputation for all 3 missing mechanisms. This result is consistent with the previous study [7].

Recent studies have used autoencoders to reduce the dimension of high-dimensional features and then calculate propensity scores using the reduced features [17]. It leverages the ability of neural networks to deal with high-dimensional data. However, they did not consider reconstruction and computation of the propensity score as joint tasks. Instead, we train the model together with reconstruction of the input, prediction of missing patterns, and estimation of propensity scores as joint tasks to prevent overfitting. It causes propensity scores to be close to zero or one, resulting in biased estimates of the effects.

As the variable dimension increases in observational studies, the relationship between variables will be more complex, and missing will be more difficult to avoid. It also becomes increasingly difficult to manually determine propensity models for high-dimensional variables. The neural network has the ability to model complex models, so there is no need to manually specify the so-called correct model, and the neural network can learn adaptively by observing the data. Multiple imputation is expensive for large datasets. In contrast, for the MTNN model, the computational cost of this process is smaller. Furthermore, Compared to multiple imputation [37], MTNN does not require any prior assumptions about the distribution of the data. It automatically learns the correlations between variables, thus impute their missing values.

In practice, a missing rate of greater than 30% is generally considered too high to make a reliable inference, but we want to thoroughly test the MTNN model’s stability and performance under different missing rate scenarios. Due to this, we have created a list of missing rates that are relatively high. We found that even when the missing rate is high, MTNN still performs well. It shows that the correlation between variables can be captured and utilized very effectively. Even though an increase in missing rates decreases the performance of the MTNN model, it still outperforms other methods.

## Limitations

Our study also has some limitations. First, there is a slight difference in performance between simulated and real data for the MTNN model. The reason for this phenomenon is that in real-world data, relationships between variables are more complex. It is difficult to simulate these unknowable complex connections manually. Due to the fact that our experiments simulate only the simplest possible case, there is a slightly different result between the 2 types of data. Second, we cannot know the true effect of real-world data. Our model aims to establish a more accurate method for estimating model parameters when missing values are present. For this purpose, a complete real data modeling process is used as a standard of evaluation. It is our goal to prove that the proposed method can estimate the parameter value with the missing value as close as possible to the parameter value estimated without the missing value. Therefore, “true effect” should actually mean “effect estimated from full data” in real-world data. Third, MTNN assumes that input variables are correlated. Using the joint learning technique and the shared hidden layer, this correlation is used to estimate propensity scores and fill in missing values. When the input variables are independent or weakly correlated, MTNN may be unable to provide accurate estimates.

## Conclusion

In this study, we propose a novel method for estimating propensity scores in data with missing values. It is based on a multi-task neural network, where missing value imputation and propensity score estimation are jointly trained as related tasks. Through the experimental results of simulated data and real-world data, we prove that our model has the smallest error in estimating the true effect under different missing mechanisms and different missing rates, and the standard deviation of the effect estimate is also the smallest. This shows that our method has good applicability in real-world observational studies with missing values.

## Supplementary Information

**Additional file 1:** **Table S1** Variable descriptions for the real dataset. **Table S2** Summary of the real dataset. **Table S3** Estimation of the true effect in the simulated datasets using three different methods under the MAR mechanism. **Table S4** Estimation of the true effect in the simulated datasets using three different methods under the MNAR mechanism. **Table S5** Estimation of the true effect in the real datasets using three different methods under the MAR mechanism. **Table S6** Estimation of the true effect in the real datasets using three different methods under the MNAR mechanism. **Table S7** Regression coefficients for real-world data without missing values. **Table S8** Spearman's correlation coefficient for each input variable in real-world data.

## Data Availability

The data in this study is available from the corresponding author on reasonable request. Readers interested in the code of the simulation analysis may contact the corresponding author.

## Footnotes

1 https://github.com/ljwa2323/MTNN.
