# Supplementary material for: Propensity score analysis with missing data using a multi-task neural network
Source: BMC Med Res Methodol. 2023 Feb 15;23:41. doi: 10.1186/s12874-023-01847-2 (PMC9930709; doi:10.1186/s12874-023-01847-2)
Supplement: Supplementary file 1 — Additional file 1: Table S1 Variable descriptions for the real dataset. Table S2 Summary of the real dataset. Table S3 Estimation of the true effect in the simulated datasets using three different methods under the MAR mechanism. Table S4 Estimation of the true effect in the simulated datasets using three different methods under the MNAR mechanism. Table S5 Estimation of the true effect in the real datasets using three different methods under the MAR mechanism. Table S6 Estimation of the true effect in the real datasets using three different methods under the MNAR mechanism. Table S7 Regression coefficients for real-world data without missing values. Table S8 Spearman's correlation coefficient for each input variable in real-world data. [file 12874_2023_1847_MOESM1_ESM.docx]

## *Supplementary Online Content*

## *S.1 The process of estimating propensity scores using MTNN*

## *Forward process of MTNN*

The process of estimating propensity scores using MTNN is divided into two stages. The first stage involves training MTNN based on covariates and grouping/exposure information so that it can learn how covariates and missing patterns relate to propensity scores. The second stage is to use the trained MTNN model to estimate the missing value of each sample and estimate the propensity score.

1. Stage one: suppose we have *n* samples, *K* covariates, $X_{ki}$ is the k-th covariate of the i-th object, and the missing status of $X_{ki}$ is represented by $M_{ki}$, where if $X_{ki}$ is missing, it is 1, otherwise it is 0. Missing values in $X_{ki}$ are replaced with the mean. In an iterative process, the common hidden layer is first calculated, $H_{1i}=\mathrm{Tanh}\left( W_{1}\left[ X_{i},M_{i} \right]+b_{1} \right)$, and then $H_{1i}$ flows to 3 sub-networks respectively. Tanh represents the hyperbolic tangent activation function, which has the form $\frac{2}{1+e^{-2x}}-1$. For reconstruction of covariates, the first subnetwork uses the input $H_{1i}$ and estimates the reconstructed covariates $\tilde{X}_{i}=W_{31}\left( W_{21}H_{1i}+b_{21} \right)+b_{31}$. For predicting treatment groups/exposure levels, the second subnetwork uses $H_{1i}$ as input and predicts the probability of missing each variable for subject i, $\tilde{M}_{i}=\sigma(W_{33} \sigma\left( W_{23}H_{1i}+b_{23} \right)+b_{33})$. The third subnetwork responsible for predicting the treatment group/exposure level takes $H_{1i}$ as input and predicts the probability that subject i belongs to the treatment group. $\tilde{T}_{i}=\sigma(W_{32} \sigma\left( W_{22}H_{1i}+b_{22} \right)+b_{32})$. Then use the three outputs from the network to construct the overall objective function.

$$\mathcal{L}_{1}=\frac{1}{nK}\sum_{i=1}^{n} \sum_{k=1}^{K} \left( X_{ki}-\tilde{X}_{ki} \right)^{2}(1-M_{ki})$$

$$\mathcal{L}_{2}=-\frac{1}{nK}\sum_{i=1}^{n} \sum_{k=1}^{K} \left( M_{i}\log\left( \tilde{M}_{i} \right)+\left( 1-M_{i} \right)\log\left( 1-\tilde{M}_{i} \right) \right)$$

$$\mathcal{L}_{3}=-\frac{1}{n}\sum_{i=1}^{n} \left( T_{i}\log\left( \tilde{T}_{i} \right)+\left( 1-T_{i} \right)\log\left( 1-\tilde{T}_{i} \right) \right)$$

$$\mathcal{L}_{total}=\lambda_{1}\mathcal{L}_{1}+\lambda_{2}\mathcal{L}_{2}+\lambda_{3}\mathcal{L}_{3}$$

In these formulas, $W_{1},W_{21},W_{22},W_{23},W_{31},W_{32},W_{33},b_{1},b_{21},b_{22},b_{23},b_{31},b_{32},b_{33}$ are the parameters to be learned by the model. They represent the weights or offsets of the model. $\mathcal{L}_{1}$, $\mathcal{L}_{2}$ and $\mathcal{L}_{3}$ represent the loss functions of the three tasks, respectively. The Adam optimizer is used to optimize the objective function $\mathcal{L}_{total}$ until the loss function converges. $\lambda_{1}$, $\lambda_{2}$, $\lambda_{3}$ are all set to 1. We set the number of layers in the common hidden layer to 1, as well as the number of layers in the branch subnetworks. Each hidden layer has 100 neurons.

1. Stage two: when the objective function has converged, the first sub-network is used to estimate and replace missing values, while the third sub-network is used to calculate the propensity score of each individual. The imputed covariates $\hat{X}$ and propensity scores $\hat{e}$ were then used for subsequent inverse probability weighted regression analyses to estimate treatment effects.

$$\hat{X}_{ki}\leftarrow X_{ki}\circ(1-M_{ki})+\tilde{X}_{ki}\circ M_{ki}$$

$$\hat{e}_{i}= \sigma\left( W_{32} \sigma\left( W_{22}\mathrm{Tanh}\left( W_{1}\left[ X_{i},M_{i} \right]+b_{1} \right)+b_{22} \right)+b_{32} \right)$$

$\circ$ represents element-wise multiplication.

## *Gradient backpropagation*

The advantage of using MTNN for propensity score estimation is that through joint learning, the correlation information between covariates, missing patterns, and propensity scores can be fully utilized. Let $\theta$ be the parameter of the public hidden layer in the MTNN model, then its gradient during the learning process can be written as:

$$\nabla_{\theta}\mathcal{L}_{total}=\lambda_{1}\frac{\partial\mathcal{L}_{1}}{\partial\theta}+\lambda_{2}\frac{\partial\mathcal{L}_{2}}{\partial\theta}+\lambda_{3}\frac{\partial\mathcal{L}_{3}}{\partial\theta}$$

Then, the calculated gradient is used to update the parameter $\theta$.

$$\theta^{'}=\theta-\eta\nabla_{\theta}\mathcal{L}_{total}$$

According to the gradient backpropagation algorithm, these parameters are updated by three related tasks. In this way, the shared hidden layer extracts features that satisfy all three optimization requirements at the same time. The learning rate during training is fixed at 0.001. All samples are used in each iteration. The maximum number of iterations is 1000.

It should be noted that the imputation of missing values through methods such as single value imputation, mean value imputation, and multiple imputation is independent of the estimation of propensity scores. At the data imputation stage, independent imputation procedures may introduce additional errors, such as relationships between covariates and treatment/exposure that do not exist. Therefore, treatment estimates may be biased. MTNN, on the other hand, views the two as a whole, which eliminates the aforementioned problems. A common hidden layer serves as a central component of MTNN, allowing information to be transferred between tasks. As a result, the process of filling in missing values and estimating propensity scores can be mutually reinforcing.

**Table S1** Variable descriptions for the real dataset.

| Variable | Description |
| --- | --- |
| treat | The treatment assignment (1=treated, 0=control). |
| age | The age in years. |
| educ | The education in number of years of schooling. |
| race | The individual's race/ethnicity.(Black, Hispanic, or White). |
| married | an indicator for married (1=married, 0=not married). |
| nondegree | The indicator for whether the individual has a high school degree (1=no degree, 0=degree). |
| re74 | The income in 1974, in U.S. dollars. |
| re75 | The income in 1975, in U.S. dollars. |
| re78 | The income in 1978, in U.S. dollars. |

**Table S2** Summary of the real dataset.

|  | Treat=0 | Treat=1 | Overall | *P* value |
| --- | --- | --- | --- | --- |
| n | 429 | 185 | 614 |  |
| age (mean (SD)) | 28.03 (10.79) | 25.82 (7.16) | 27.36 (9.88) | 0.011 |
| educ (mean (SD)) | 10.24 (2.86) | 10.35 (2.01) | 10.27 (2.63) | 0.633 |
| race (%) |  |  |  | <0.001 |
| black | 87 (20.3) | 156 (84.3) | 243 (39.6) |  |
| hispan | 61 (14.2) | 11 ( 5.9) | 72 (11.7) |  |
| white | 281 (65.5) | 18 ( 9.7) | 299 (48.7) |  |
| married (%) | 220 (51.3) | 35 (18.9) | 255 (41.5) | <0.001 |
| nondegree (%) | 256 (59.7) | 131 (70.8) | 387 (63.0) | 0.011 |
| re74 (mean (SD)) | 5619.24 (6788.75) | 2095.57 (4886.62) | 4557.55 (6477.96) | <0.001 |
| re75 (mean (SD)) | 2466.48 (3292.00) | 1532.06 (3219.25) | 2184.94 (3295.68) | 0.001 |
| re78 (mean (SD)) | 6984.17 (7294.16) | 6349.14 (7867.40) | 6792.83 (7470.73) | 0.334 |

SD, standard deviation

**Table S3** Estimation of the true effect in the simulated datasets using three different methods under the MAR mechanism.

| Missing rate | Method | True effect =0 | | |  | True effect =1 | | |
| --- | --- | --- | --- | --- | --- | --- | --- | --- |
|  |  | Mean | SD | RMSE |  | Mean | SD | RMSE |
| 0.2 | Missing indicator | 0.111 | 0.043 | 0.119 |  | 1.111 | 0.043 | 1.112 |
|  | Multiple imputation | 0.118 | 0.047 | 0.126 |  | 1.103 | 0.065 | 1.105 |
|  | Multi-task neural network | 0.088 | 0.048 | 0.099 |  | 1.086 | 0.047 | 1.087 |
| 0.3 | Missing indicator | 0.16 | 0.057 | 0.169 |  | 1.16 | 0.057 | 1.161 |
|  | Multiple imputation | 0.163 | 0.081 | 0.18 |  | 1.162 | 0.066 | 1.163 |
|  | Multi-task neural network | 0.133 | 0.051 | 0.141 |  | 1.132 | 0.058 | 1.133 |
| 0.4 | Missing indicator | 0.165 | 0.048 | 0.171 |  | 1.165 | 0.048 | 1.166 |
|  | Multiple imputation | 0.177 | 0.051 | 0.184 |  | 1.178 | 0.052 | 1.179 |
|  | Multi-task neural network | 0.121 | 0.044 | 0.128 |  | 1.123 | 0.047 | 1.124 |
| 0.5 | Missing indicator | 0.205 | 0.057 | 0.212 |  | 1.205 | 0.057 | 1.206 |
|  | Multiple imputation | 0.202 | 0.076 | 0.214 |  | 1.215 | 0.058 | 1.217 |
|  | Multi-task neural network | 0.179 | 0.053 | 0.186 |  | 1.174 | 0.067 | 1.176 |
| 0.6 | Missing indicator | 0.22 | 0.047 | 0.225 |  | 1.22 | 0.047 | 1.221 |
|  | Multiple imputation | 0.238 | 0.054 | 0.243 |  | 1.239 | 0.067 | 1.241 |
|  | Multi-task neural network | 0.183 | 0.046 | 0.188 |  | 1.188 | 0.048 | 1.189 |
| 0.7 | Missing indicator | 0.249 | 0.077 | 0.259 |  | 1.249 | 0.077 | 1.251 |
|  | Multiple imputation | 0.24 | 0.067 | 0.248 |  | 1.239 | 0.065 | 1.241 |
|  | Multi-task neural network | 0.211 | 0.055 | 0.217 |  | 1.211 | 0.058 | 1.212 |
| 0.8 | Missing indicator | 0.27 | 0.068 | 0.277 |  | 1.27 | 0.068 | 1.271 |
|  | Multiple imputation | 0.256 | 0.065 | 0.263 |  | 1.252 | 0.068 | 1.253 |
|  | Multi-task neural network | 0.216 | 0.051 | 0.221 |  | 1.225 | 0.058 | 1.226 |

SD, standard deviation; RMSE, root mean square error.

**Table S4** Estimation of the true effect in the simulated datasets using three different methods under the MNAR mechanism.

| Missing rate | Method | True effect=0 | | |  | True effect=1 | | |
| --- | --- | --- | --- | --- | --- | --- | --- | --- |
|  |  | Mean | SD | RMSE |  | Mean | SD | RMSE |
| 0.2 | Missing indicator | 0.105 | 0.047 | 0.114 |  | 1.105 | 0.047 | 1.106 |
|  | Multiple imputation | 0.135 | 0.070 | 0.150 |  | 1.127 | 0.053 | 1.128 |
|  | Multi-task neural network | 0.087 | 0.073 | 0.110 |  | 1.08 | 0.046 | 1.081 |
| 0.3 | Missing indicator | 0.124 | 0.047 | 0.132 |  | 1.124 | 0.047 | 1.125 |
|  | Multiple imputation | 0.159 | 0.069 | 0.172 |  | 1.168 | 0.071 | 1.17 |
|  | Multi-task neural network | 0.083 | 0.031 | 0.088 |  | 1.08 | 0.042 | 1.081 |
| 0.4 | Missing indicator | 0.149 | 0.055 | 0.158 |  | 1.149 | 0.055 | 1.150 |
|  | Multiple imputation | 0.180 | 0.078 | 0.194 |  | 1.198 | 0.072 | 1.200 |
|  | Multi-task neural network | 0.143 | 0.063 | 0.155 |  | 1.126 | 0.068 | 1.128 |
| 0.5 | Missing indicator | 0.162 | 0.066 | 0.174 |  | 1.162 | 0.066 | 1.164 |
|  | Multiple imputation | 0.214 | 0.064 | 0.223 |  | 1.226 | 0.072 | 1.227 |
|  | Multi-task neural network | 0.143 | 0.057 | 0.152 |  | 1.143 | 0.064 | 1.144 |
| 0.6 | Missing indicator | 0.215 | 0.077 | 0.227 |  | 1.215 | 0.077 | 1.217 |
|  | Multiple imputation | 0.236 | 0.079 | 0.247 |  | 1.24 | 0.066 | 1.242 |
|  | Multi-task neural network | 0.171 | 0.055 | 0.178 |  | 1.185 | 0.065 | 1.187 |
| 0.7 | Missing indicator | 0.24 | 0.091 | 0.254 |  | 1.24 | 0.091 | 1.243 |
|  | Multiple imputation | 0.245 | 0.084 | 0.257 |  | 1.255 | 0.085 | 1.258 |
|  | Multi-task neural network | 0.205 | 0.075 | 0.216 |  | 1.216 | 0.084 | 1.218 |
| 0.8 | Missing indicator | 0.229 | 0.082 | 0.241 |  | 1.229 | 0.082 | 1.231 |
|  | Multiple imputation | 0.259 | 0.074 | 0.268 |  | 1.258 | 0.068 | 1.260 |
|  | Multi-task neural network | 0.210 | 0.059 | 0.217 |  | 1.219 | 0.061 | 1.221 |

SD, standard deviation; RMSE, root mean square error.

**Table S5** Estimation of the true effect in the real datasets using three different methods under the MAR mechanism.

| Missing rate | Method | Mean | SD | RMSE |
| --- | --- | --- | --- | --- |
| 0.2 | Missing indicator | 324.632 | 245.564 | 451.919 |
|  | Multiple imputation | 517.256 | 301.065 | 344.651 |
|  | Multi-task neural network | 752.46 | 218.154 | 209.478 |
| 0.3 | Missing indicator | 396.622 | 146.788 | 345.087 |
|  | Multiple imputation | 530.224 | 294.751 | 332.473 |
|  | Multi-task neural network | 721.581 | 190.617 | 179.933 |
| 0.4 | Missing indicator | 375.492 | 243.591 | 408.022 |
|  | Multiple imputation | 292.261 | 169.244 | 449.74 |
|  | Multi-task neural network | 697.548 | 164.66 | 155.984 |
| 0.5 | Missing indicator | 244.378 | 249.551 | 524.139 |
|  | Multiple imputation | 425.151 | 181.431 | 334.617 |
|  | Multi-task neural network | 708.351 | 120.458 | 113.654 |
| 0.6 | Missing indicator | 386.029 | 164.392 | 361.613 |
|  | Multiple imputation | 398.418 | 113.207 | 331.952 |
|  | Multi-task neural network | 636.049 | 157.032 | 166.736 |
| 0.7 | Missing indicator | 364.815 | 96.159 | 359.545 |
|  | Multiple imputation | 453.507 | 138.367 | 290.209 |
|  | Multi-task neural network | 675.976 | 159.393 | 154.71 |
| 0.8 | Missing indicator | 342.044 | 192.954 | 412.931 |
|  | Multiple imputation | 428.794 | 167.997 | 325.137 |
|  | Multi-task neural network | 608.742 | 135.768 | 164.927 |

SD, standard deviation; RMSE, root mean square error.

**Table S6** Estimation of the true effect in the real datasets using three different methods under the MNAR mechanism.

| Missing rate | Method | Mean | SD | RMSE |
| --- | --- | --- | --- | --- |
| 0.2 | Missing indicator | 349.65 | 288.126 | 453.463 |
|  | Multiple imputation | 403.597 | 202.79 | 363.491 |
|  | Multi-task neural network | 768.091 | 76.024 | 90.558 |
| 0.3 | Missing indicator | 357.472 | 221.402 | 412.055 |
|  | Multiple imputation | 490.795 | 256.025 | 327.912 |
|  | Multi-task neural network | 764.515 | 211.965 | 206.44 |
| 0.4 | Missing indicator | 419.709 | 237.371 | 368.718 |
|  | Multiple imputation | 281.221 | 331.057 | 532.572 |
|  | Multi-task neural network | 680.861 | 191.843 | 183.659 |
| 0.5 | Missing indicator | 374.335 | 194.206 | 384.767 |
|  | Multiple imputation | 303.363 | 217.669 | 457.938 |
|  | Multi-task neural network | 679.976 | 146.858 | 142.283 |
| 0.6 | Missing indicator | 349.170 | 117.998 | 380.212 |
|  | Multiple imputation | 437.073 | 325.978 | 412.854 |
|  | Multi-task neural network | 632.000 | 239.189 | 239.529 |
| 0.7 | Missing indicator | 370.742 | 107.923 | 356.816 |
|  | Multiple imputation | 298.889 | 160.822 | 440.755 |
|  | Multi-task neural network | 639.842 | 127.069 | 140.239 |
| 0.8 | Missing indicator | 527.317 | 306.97 | 343.72 |
|  | Multiple imputation | 263.805 | 137.503 | 467.281 |
|  | Multi-task neural network | 695.674 | 114.39 | 109.19 |

SD, standard deviation; RMSE, root mean square error.

**Table S7** Regression coefficients for real-world data without missing values

|  | $\hat{\beta}$ | Std. Error | t value | Pr(>\|t\|) |
| --- | --- | --- | --- | --- |
| (Intercept) | -1403.15 | 2551.65 | -0.55 | 0.58 |
| treat | 712.74 | 552.51 | 1.29 | 0.20 |
| age | 22.69 | 33.26 | 0.68 | 0.50 |
| educ | 440.72 | 167.84 | 2.63 | 0.01 |
| race (hispan) | 606.79 | 910.86 | 0.67 | 0.51 |
| race (white) | 958.64 | 610.18 | 1.57 | 0.12 |
| married | -433.4 | 654.41 | -0.66 | 0.51 |
| nodegree | 750.6 | 834.89 | 0.90 | 0.37 |
| re74 | 0.20 | 0.06 | 3.34 | <0.001 |
| re75 | 0.44 | 0.11 | 4.07 | <0.001 |

**Table S8** Spearman's correlation coefficient for each input variable in real-world data

|  | treat | age | educ | race(hispan) | race(white) | married | nodegree | re74 |
| --- | --- | --- | --- | --- | --- | --- | --- | --- |
| age | -0.03 |  |  |  |  |  |  |  |
| educ | -0.01 | 0.05 |  |  |  |  |  |  |
| race(hispan) | -0.12 | -0.04 | -0.15 |  |  |  |  |  |
| race(white) | -0.51 | 0.11 | 0.12 | -0.36 |  |  |  |  |
| married | -0.30 | 0.44 | -0.05 | 0.02 | 0.30 |  |  |  |
| nodegree | 0.11 | -0.15 | -0.85 | 0.10 | -0.17 | -0.03 |  |  |
| re74 | -0.37 | 0.30 | 0.12 | 0.03 | 0.32 | 0.43 | -0.21 |  |
| re75 | -0.23 | 0.13 | 0.04 | 0.08 | 0.19 | 0.31 | -0.08 | 0.63 |
